# Enigma of categorizing COVID-19-related lung parenchymal diseases and management experience with follow-up outcomes in Qatar: a case series

**DOI:** 10.5339/qmj.2022.2

**Published:** 2022-02-18

**Authors:** Phool Iqbal, Muhammad Bilal Jamshaid, Aamir Shahzad, Zohaib Yousaf, Saba Nabavi Monfared, Nagham D. Sadik, Ibrahim Fawzy Hassan

**Affiliations:** ^1^Internal Medicine Department, Hamad Medical Corporation, Doha, Qatar E-mail: dr.phooliqbal89@gmail.com; ^2^Critical Care Department, Hamad Medical Corporation, Doha, Qatar; ^3^Reading Hospital, Tower health, Pennsylvania, USA; ^4^Dresden International University, Dresden, DEU, Germany; ^5^Trauma & Emergency Clinical Pharmacist Department, Hamad Medical Corporation, Doha, Qatar; ^6^Pulmonology and critical care department, Hamad Medical Department, Doha, Qatar

**Keywords:** Medicine, critical care, COVID-19, lung parenchymal diseases

## Abstract

Coronavirus disease 2019 (COVID-19) has become one of the worst global pandemics in recent history. Post-COVID-19 interstitial lung disease is a significant concern in COVID-19 survivors. It is a disabling clinical condition for patients and a burden on the healthcare system. With time and subsequent waves of COVID-19 globally, the post-COVID-19 sequelae of lung diseases can be debilitating. We report cases of three patients with persistent hypoxia post-COVID-19, raising concerns for interstitial lung disease in Qatar. In this report, we shared our experience of the patient's clinical course, complications, and outcomes with post-COVID-19 sequelae of lung parenchymal disease. Patients were followed up during and after treatment until recovery or discharge from the hospital.

## Introduction

The novel coronavirus disease 2019 (COVID-19) primarily affects the respiratory system and commonly presents with cough, fever, myalgias, and shortness of breath. Chest X-ray (CXR) and computed tomography (CT) of the lungs may reveal consolidation, fibrotic pattern, or ground-glass opacities.^1^ As more patients recover from COVID-19, there is emerging evidence of post-COVID-19-related interstitial lung disease, raising concerns of increased morbidity and mortality in its survivors.^2,3^ Every country has developed management strategies based on available resources and local guidelines along with standard mass vaccination. Herein, we present a case series of patients with COVID-19 during the first and second waves in Qatar, who presented with persistent hypoxia, and its possible management strategy based on the literature review. This case report is worthy of publication, as it highlights various clinical signs and further prognostication upon follow-ups.

## Case Presentation

### Case 1

A 40-year-old healthy gentleman presented with fever, cough, and shortness of breath for 2 days with tachypnea of approximately 30/min and requiring 5 L/min of oxygen (O_2_) supplementation through a face mask. His CXR revealed bilateral pulmonary infiltrates, and he was diagnosed with COVID-19 by real-time reverse-transcriptase polymerase chain reaction (rRT-PCR) from a nasopharyngeal swab. He had COVID-19-associated severe pneumonia at presentation and was intubated. He received tocilizumab and methylprednisolone intravenously due to a suspected cytokine storm. He was extubated after 2 weeks, and 6 weeks post-extubation, he had persistent hypoxia at rest requiring O_2_ supplementation of 4–5 L/min through a facemask. He was further examined, and possible causes of hypoxia, including any heart diseases (excluded by echocardiography) and secondary infections such as bacterial, fungal, and tuberculosis, were ruled out. A high-resolution chest CT showed extensive ground-glass opacities at the peripheral and subpleural regions with fibrotic changes (Figure 1a, blue arrows) correlating with COVID-19-related pulmonary fibrosis leading to interstitial lung disease. He was commenced on a short-course therapy of oral 40 mg prednisolone daily with tapering dose over 3 weeks and received pulmonary rehabilitation therapy. He responded well to all measures and was discharged on day 64. He was followed as an outpatient for 2 months. He was successfully weaned off from O_2_ therapy and remained asymptomatic during his follow-up; however, later, he traveled back to his country.

### Case 2

A 46-year-old gentleman with a past medical history of type 2 diabetes mellitus, hypertension, and end-stage renal disease post-renal graft rejection presented to the hospital with fever, myalgia, and shortness of breath. He presented during the second wave of COVID-19 and tested positive for severe acute respiratory syndrome coronavirus 2 (SARS-CoV-2) RT-PCR from a nasopharyngeal swab. He had complicated severe COVID-19 pneumonia that required endotracheal intubation and mechanical ventilation. He was managed as per local guidelines and received remdesivir, methylprednisolone, tocilizumab, and anakinra intravenously for COVID-19-associated acute respiratory distress syndrome (ARDS). He was tracheostomized after 1 month because of difficulty weaning off from mechanical ventilation. He had fibrotic changes in the lung parenchyma (Figure 1b). He underwent pulmonary rehabilitation therapy. He started to show some improvements after 2 months with negative SARS-CoV-2 PCR, underwent pulmonary rehabilitation therapy, and received 40 mg prednisolone daily with tapering dose over 4 weeks. Eventually, he underwent tracheostomy closure and did not require O_2_ supplementation. The patient is currently admitted and is followed by the rehabilitation team for critical care myopathy.

### Case 3

A 48-year-old gentleman without any past medical history presented to the hospital with fever, cough, headache, nausea, abdominal pain, and shortness of breath for 5 days. He tested positive for COVID-19 RT-PCR during the second wave of the COVID-19. He developed tachypnea of 32–34/min requiring O_2_ supplementation through high-flow nasal cannula with fraction of inspired O_2_ of 60% and O_2_ at flow rate of 60 L/min. CT pulmonary angiogram ruled out pulmonary embolism. He was treated with azithromycin, remdesivir, methylprednisolone, tocilizumab, and anakinra intravenously as per the local guidelines for COVID-19-related ARDS. He did not require endotracheal intubation. However, he had persistent hypoxia up to 6 weeks post-negative SARS-CoV-2 PCR. His chest CT showed extensive fibrotic changes coinciding with COVID-19-associated interstitial lung disease (Figure 1c). He underwent pulmonary rehabilitation therapy, was kept on methylprednisolone 60 mg intravenously twice daily for 2 weeks, and was switched to oral prednisolone 40 mg with tapering dose. He was commenced on pirfenidone 267 mg (anti-fibrotic agent) three times daily and for further follow-up with a pulmonologist. After 3 weeks of therapy, his condition improved and is currently on O_2_ supplementation of 3–5 L/min through a face mask and can also mobilize. The patient was treated in the intensive care unit and was then moved to the medical floor. He was discharged after 1 month with the requirement of O_2_ supplementation of 1–2 L through a nasal cannula on ambulation.

## Discussion

COVID-19 has become one of the worst economic, social, and healthcare disasters in recent global history. Various mutant variants of the virus are emerging, with differences in infectivity and transmissibility rate. B.1.1.7 (UK variant), B.1.351 (South African variant), and P.1 (Brazil/Japan variant) have been detected recently in the initial phase of the second wave and were reported to have high transmissibility, infectivity, and even evade COVID-19 vaccines.^4^ In the post-COVID-19 sequelae of recovered cases, more than a third of the patients are at risk of developing fibrotic lung function abnormalities. Moreover, 47% of the patients had impaired gas transfer measured by the diffusing capacity of the lungs for carbon monoxide and 25% had reduced total lung capacity.^5^ Post-COVID-19 follow-up studies of the survivors have revealed acute “fibrotic-like” changes in the lungs, which resolved, while some of them may have persistent fibrotic changes that lead to interstitial lung disease.^6^ Patients with pulmonary fibrosis experience fatigue, shortness of breath, and dry cough, resulting in decreased functional capacity and poor quality of life.^5,7^

Similar case reports have raised the concerns of post-COVID-19 lung fibrosis that affects the quality of life, increases long-term healthcare burden, and thus affects overall morbidity.^8–12^ Suggested mechanisms include cytokine storm, systemic inflammation leading to alveolar wall damage, drug-induced pulmonary toxicity, high airway pressure associated with mechanical ventilation, and hyperoxia-induced lung injury leading to lung damage.^5,12^ Factors such as advanced age, multiple comorbidities, disease severity, prolonged mechanical ventilation, smoking history, and chronic alcoholism are also high-risk factors.^13^

Considering the underlying systemic inflammation and cytokine release cascade in post-COVID-19 pulmonary fibrosis, steroids, interleukin-6 inhibitors, interleukin-1 inhibitors such as anakinra, antifibrotic agents such as nintedanib, pirfenidone, and investigational humanized monoclonal antibody drugs such as sarilumab and canakinumab are being considered.^14–17^

In our case series of patients in Qatar, we have compared the patient's demography, clinical presentation, CT chest findings, and hospital course with management, as shown in Table 1.

Certain aspects are highlighted based on the outcomes of our patients. Fibrotic-like changes are more likely to resolve compared with “post-COVID-19 fibrosis.”^6^ In our first case, the patient had post-COVID-19 lung parenchymal disease following recovery from COVID-19. On the contrary, the second and third patients had fibrotic-like changes beyond the acute phase of COVID-19 and were most likely to be in the recovery phase. These patients responded well to the supportive therapy including chest physiotherapy and rehabilitation therapy, respectively, apart from the local treatment pathway. The third patient was discharged on pirfenidone considering the beneficial role of antifibrotic therapy in lung fibrosis in patients with COVID-19; however, further studies are required to consolidate this finding. There is a fine line between the two clinical conditions, and only follow-up can segregate both conditions based on resolution compared with O_2_ dependency in the long-term.^8^ Pulmonary rehabilitation therapy and regular follow-ups are required in such patients.^7,18^ Various proposals have been suggested based on consensus and expert opinion of the scientific society for regular follow-ups post-discharge at certain time intervals such as 3 and 6 months or 4–12 weeks, ≥ 12 weeks for symptomatic COVID-19 survivors with diagnostic modalities such as CT scan, pulmonary function tests, and diffusion capacity of the lungs to target and manage such category of patients earlier in the disease progression.^8,19,20^ However, currently, there are no management guidelines, and further studies with long-term follow-ups are required in COVID-19 and post-COVID-19 lung parenchymal disease cases for categorization and management accordingly.

## Conclusion

As we are heading toward different waves of COVID-19 globally, it is expected to see numerous post-COVID-19 sequelae of lung parenchymal disease. A new entity of post-COVID-19 interstitial lung disease may be introduced. It will be a future challenge that may require further criteria and categorization for management. We strongly advise close follow-up at regular intervals and monitoring of such patients through pulmonary function tests and high-resolution CT to monitor changes in lung anatomy and function. Controlling modifiable risk factors such as smoking cessation, obesity hypertension, and diabetes control can also help in this regard.

### Conflict of interest/Disclosure statement

The authors certify that they have no conflict of interest and no affiliations with or involvement in any organization or entity with any financial or non-financial interest in the subject matter or materials discussed in this manuscript.

### Patient consent

Each patient has given informed consent to publish the case.

### Funding Sources

The report was funded by the Hamad Medical Corporation, Qatar.

## Figures and Tables

**Figure 1a.**
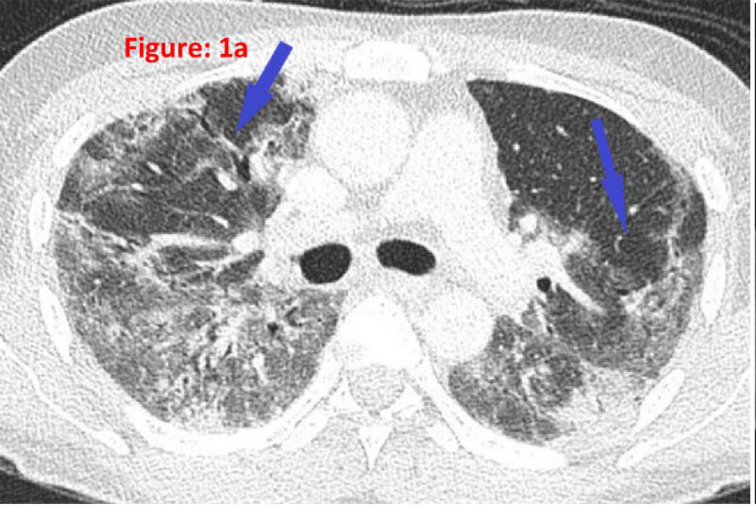
High-resolution chest computed tomography (HRCT) showing extensive ground-glass opacities at the peripheral and subpleural regions with fibrotic changes (blue arrows).

**Figure 1b.**
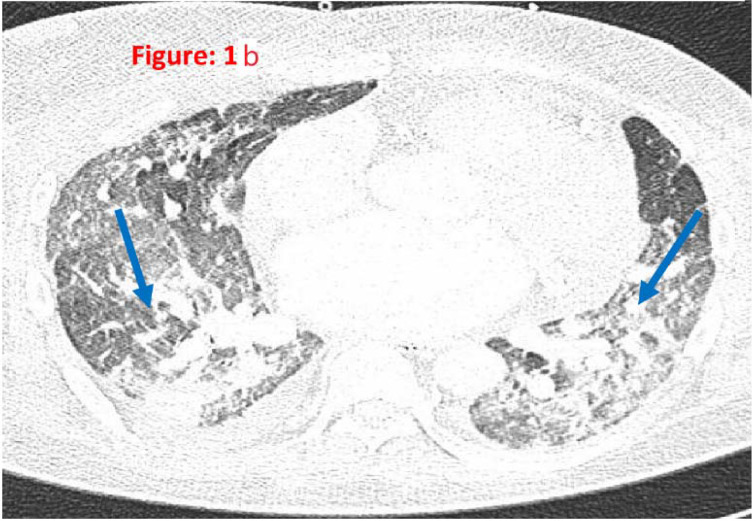
HRCT findings suggestive of diffuse fibrotic changes in the lung parenchyma (blue arrows).

**Figure 1c.**
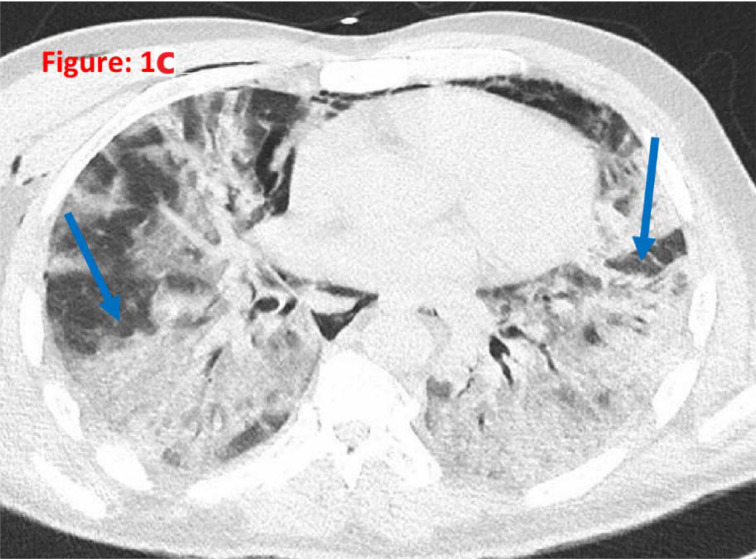
CT chest showing extensive fibrosis of the lung parenchyma and subpleural fibrosis, findings coinciding with COVID-19-associated interstitial lung disease.

**Table 1:** Patient's demography, clinical presentation, CT chest comparisons, and hospital course with management

Cases	COVID-19 wave	Age	Nationality	Oxygen requirements at presentation	Chest CT findings	Duration of stay	Treatment	Follow-up clinical status

1	1st wave	40 years	Indian	Mechanical ventilation	Interstitial thickening at the lower lobes, with septal fibrosis and ground-glass opacities	63 days, 20 days under ICU care	Tocilizumab and steroids administered intravenously, post-extubation steroids given orally, and pulmonary rehabilitation therapy	2 months, off oxygen; however, afterward traveled back to his country

3	2nd wave	46 years	Filipino	Mechanical ventilation	Ground-glass opacities involving the peripheries and bases of the lungs with bronchiectatic changes and honeycombing patterns in evolution	Still admitted	Tocilizumab, steroids, and anakinra given intravenously, post-extubation steroids given orally, and pulmonary rehabilitation	Post tracheostomy closure, stable and on oral steroids with pulmonary rehabilitation therapy

4	2nd wave	48 years	Egyptian	High-flow nasal cannula with 60% fraction of inspired oxygen	Diffuse ground-glass opacities and diffuse parenchymal involvement with fibrosis and septal thickening with alveolar wall destruction correlating with post-COVID-19 interstitial lung disease	3 months of hospital stay	Tocilizumab, steroids, and anakinra given intravenously, post-extubation steroids given orally with tapering and pirfenidone initiated	Discharged on nasal cannula 1–2 L/min oxygen and on pirfenidone


## References

[10] https://www.emjreviews.com/respiratory/article/post-covid-19-pulmonary-fibrosis-report-of-two-cases.

